# Modified Oesophago-Gastric Dissociation (M-OGD) — a technical modification

**DOI:** 10.1007/s13304-020-00934-z

**Published:** 2020-12-03

**Authors:** Riccardo Coletta, Elisa Mussi, Adrian Bianchi, Antonino Morabito

**Affiliations:** 1 Department of Pediatric Surgery, Meyer Children’s Hospital, University of Florence, Viale Gaetano Pieraccini, 24, 50139 Florence, FI Italy; 2 School of Environment and Life Science, University of Salford, Salford, UK; 3 Department of Industrial Engineering, University of Florence, via di Santa Marta, 3, 50139 Florence, Italy; 4 Royal Manchester Children’s Hospital, Manchester University NHS Foundation Trust, Manchester, UK; 5 Department of Neurofarba, University of Florence, Viale Pieraccini 6, 50121 Florence, Italy

**Keywords:** Gastroesophageal reflux, Neurodisability, Neurological impairment

## Abstract

Adhesions and fibrosis following failed primary surgery for severe gastro-oesophageal reflux (GOR) in neurologically impaired children (NI) can render mobilization of the lower oesophagus and oesophago-jejunal anastomosis a technically demanding exercise both at open surgery and laparoscopy. This paper presents the Modified Oesophago-Gastric Dissociation (M-OGD) as a less complex technical modification of the original Total Oesophago-Gastric Dissociation (TOGD). The stomach is detached from the oesophago-gastric junction with an articulated 5-mm stapler, leaving a 5-mm strip of stomach attached to the oesophagus. An end-to-side isoperistaltic oesophago-jejunostomy is created between the gastric stump and the isoperistaltic jejunal Roux loop. A jejuno-jejunal anastomosis restores bowel continuity. Between May 2018 and February 2020, M-OGD was performed on 3 NI patients with a weight of 9–27.3 kg (median = 14 kg). Median age at surgery was 60 months (18–180), median surgical time 170 min (146–280), median re-feeding time was 3 days (2–5), and median length of stay was 20 days (11–25). All patients healed primarily and after a median follow-up of 3 months, there were no problems related to the oesophago-jejunal anastomosis. M-OGD reduces the difficulties of redo oesophageal surgery following failed anti-reflux procedures, with a safer oesophago-jejunal anastomosis and a good long-term outcome.

## Introduction

In 1997, Bianchi [1] presented Total Oesophago-Gastric Dissociation (TOGD) as a definitive procedure in neurologically impaired children (NI), for permanent resolution of severe gastro-oesophageal reflux (GOR).

Although recommended as a primary procedure, most surgeons still only consider TOGD as a ‘rescue’ procedure for the recurrence of severe GOR after failed first surgery, because of the adhesions and fibrosis that can render mobilization of the lower oesophagus and the oesophago-jejunal anastomosis technically demanding during open or laparoscopic surgery. Anticipation of such potential difficulties has restricted TOGD to specialist hands in Tertiary Centres. The Modified Oesophago-Gastric Dissociation (M-OGD) offers a technically easier and reproducible solution that avoids the major difficulties of para-oesophageal surgery and a safe high oesophago-jejunal anastomosis.

### Patient  characteristics

We reviewed our experience with M-OGD from May 2018 through February 2020. The Medical Ethical Review Board of our institution stated that this study is based on information routinely collected during normal clinical care, no additional data were collected for the purposes of the study, and no intervention was given solely for the purposes of the study. Therefore, institutional review board approval was waived. Informed consent was taken for all patient in respect of national law. Three NI patients with congenital problems (Table 1) were referred by an independent gastroenterologist who classified them as having moderate/severe reflux, who suffered from recurrent aspiration events, failure to thrive, failure of gastro-jejunal or jejunal tube feeds, or who had documented recurrence of reflux after a fundoplication. Two patients had severe scoliosis and one other had a previous failed Nissen fundoplication. One patient fed by gastrostomy and the other two had a permanent naso-gastric (NG) feeding tube. None had fed orally for the 12 months prior to surgery. All three patients had marked sialorrhea and suffered recurrent severe chest infections, and one needed cardiopulmonary resuscitation during a previous hospital admission. The median age at surgery was 60 months (18–180) and the patients weighed between 9 kg and 27.3 kg (median 14 kg).

**Table 1:** Patients preoperative characteristics at surgical assesment

Patient	Sex	Congenital problems	Hip subluxation	Scoliotic deviation	Nutrition	Sialorrhea
1	M	West syndrome	Right	Right-convex—dorsal Left convex—lumbar	Naso-gastric feeding	Yes
2	M	De novo heterozygosity (c.1936A>G) of the GRIN2A gene	None	None	Naso-gastric feeding	Yes
3	M	Two homoplasmic variants in the MT-ND2 and MT–TW genes	Tight	Left convex—dorsal Left convex—lumbar	Gastrostomy	Yes

### Surgical techniques

The patients had a 24-h bowel preparation before surgery. Operation was undertaken under general anaesthesia and muscle relaxation with the patient in an anti-Trendelenburg position. The upper abdominal cavity was opened by a midline incision from the xyphoid process to the umbilicus and the left lobe of the liver was retracted medially. Any residual fundoplication was unravelled, thereby restoring normal gastro-oesophageal anatomy. If intact vagus nerves (particularly the left vagus) could not be guaranteed, a pyloroplasty was considered to avoid postoperative delayed gastric emptying.

**Fig. 1:**
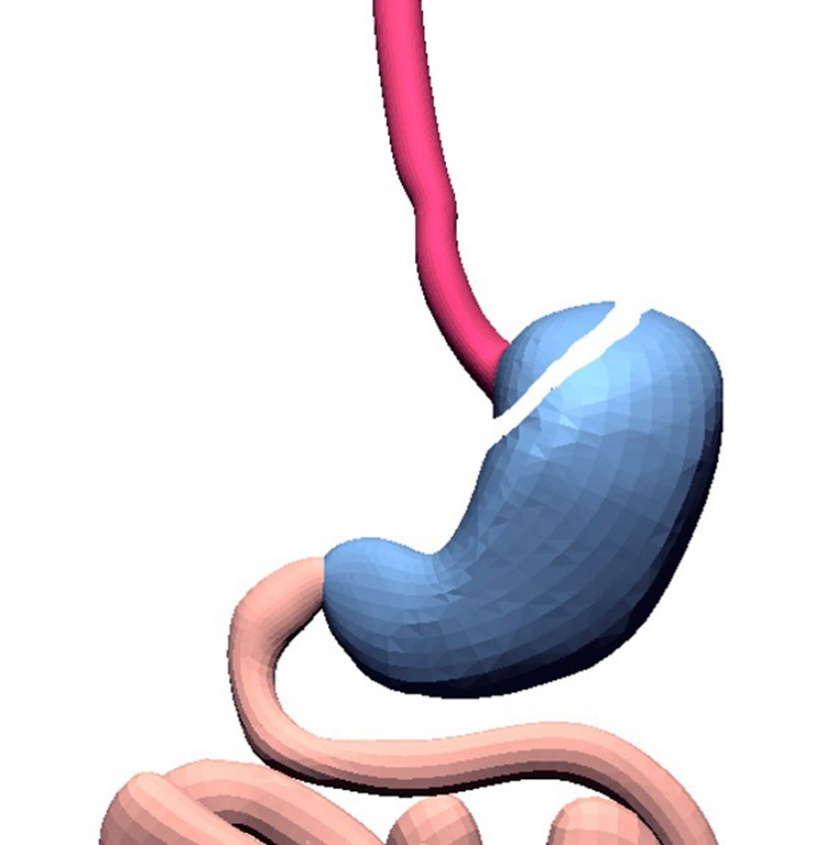
Creation of the  oesophageal-gastric stump. The gastrich stump is divided from the stomach using 5-mm Endo-GIA. Oesophagus, stomach and bowel are represented in red, blue and pink respectively

Following adequate mobilization of the oesophago-gastric junction, an articulated 5 mm laparoscopic Endo-GIA stapler (Medtronic, Endo GIA™ Ultra—code: EGIAUSHORT and Endo GIA™ Curved Tip Reload with Tri-Staple™ Technology—code: SIG45CTAVM) was applied to the stomach at 5 mm below the cardia, passing from the lesser curvature to the fundus, and the stomach transected leaving a 5 mm gastric strip attached to the distal end of the oesophagus (Fig. 1).

**Fig. 2:**
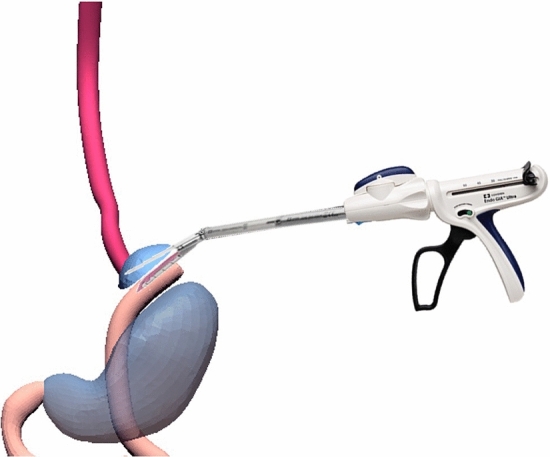
Mechanical anastomosis between the oesophago-gastric stump and isoperistaltic jejunal Roux Loop. Oesophagus, stomach and bowel are represented in red, blue and pink respectively

The proximal jejunum was transected with an Endo-GIA stapler (Medtronic, Endo GIA™ Ultra—code: EGIAUSHORT and Endo GIA™ Curved Tip Reload with Tri-Staple™ Technology—code: SIG30CTAVM) at approximately 20 cm distal to the ligament of Treitz. The distal jejunum was developed into an isoperistaltic jejunal Roux loop on a convenient and tension-free vascular pedicle that was passed through a window in the transverse mesocolon and behind the stomach to reach the oesophagus at the lesser curve. One arm of the articulated stapler was passed into the lumen of the open jejunal loop, and the opposing arm was passed into the distal oesophagus though an enterotomy at 1 cm above stapled gastric cuff such that, on firing the stapler, a 4cm end-to-side oesophago-jejunal anastomosis was created (Fig. 2). A naso-jejunal tube was positioned through the oesophago-jejunal anastomosis for free drainage of the distal jejunal Roux loop, and an abdominal drain was placed close to the anastomosis.

**Fig. 3:**
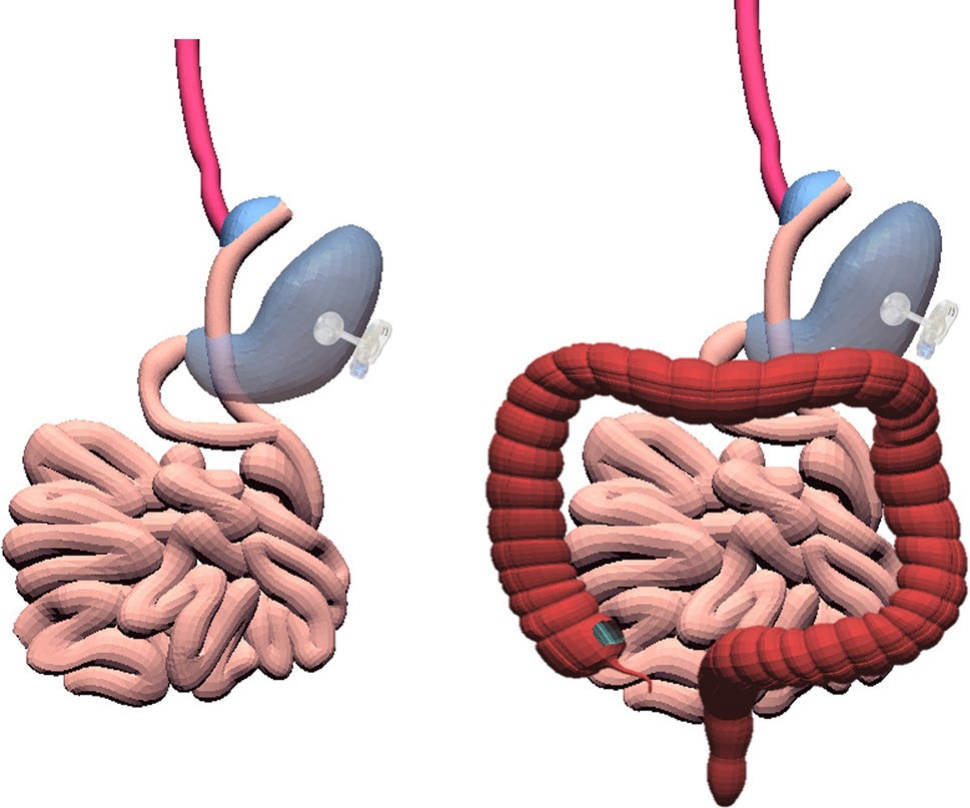
Final view of Modified Oesophago-Gastric Dissociation (M-OGD). The picture shows final gastrointestinal continuity after M-OGD. The tension-free jejunal Roux loop is passed through a window in the transverse mesocolon and behind the stomach to reach the oesophagus at the lesser curve. Oesophagus, stomach, small bowel and colon are represented in red, blue, pink and brown respectively

Bowel continuity was restored by jejuno-jejunal anastomosis at 30 – 40 cm distal to the oesophago-jejunal anastomosis, and all mesenteric defects, including the mesocolic window, were closed with interrupted sutures. Figure 3 shows the completed M-OGD with a functional gastrostomy that was retained or newly constructed (Fig. 3). Pyloroplasty was not done because of preoperative radiological evidence of good gastric emptying.

Patients were nursed in Paediatric Intensive Care for 24 h postoperatively, with free gravity drainage of both the naso-jejunal tube and the abdominal drain until return of bowel function. Any essential medication was administered via gastrostomy during the first 24hrs after surgery.

## Results

The median surgical time was 170 min (146–280), the median anaesthetic time, was 263 min (240–340), the median IOT intubation post-surgery was for 24 h (20– 26), with a median of 4 days (2–5) in the high-dependency unit. The median time to enteral feeding was 3 days (2–5), and the median length of stay was 20 days (11–25). All patients healed well without any complications at the oesophago-jejunal and the jejuno-jejunal anastomoses. During the follow-up period (65 days: range 63–101), none of the patients required hospitalization.

No complications were reported at the first outpatient follow–up visit, 1 month after discharge from hospital. At the 3-month review, one patient reported a small granuloma at the gastrostomy site. Specifically, there was no further reflux and no hospitalization episodes for respiratory problems. Sialorrhea and coughing were markedly reduced and were no longer regarded as problematic. All three patients were tolerating gastrostomy nutrition and, for the first-time, two showed an interest in oral drinks. The three families commented on the happiness of the children since operation.

## Comments

Neurologically impaired children have a high incidence of severe GOR with frequent episodes of aspiration pneumonia and hospitalization. Many fail medical management and are referred for a feeding gastrostomy and for antireflux surgery. A compiled fundoplication failure rate for neurologically impaired patients is reported to be 12–45% for primary and 20–28% following redo fundoplication [2, 3]. These failures led to a redo fundoplication rate of 6–14% [4].

TOGD has been used more commonly in Europe [5–7], but there are only limited reports for this approach in the United States [8, 9] or other non-EU [10, 11] countries. However, for a very specific and complex patient population, this procedure has a high likelihood of permanently eliminating recurrent reflux and aspiration, and of improving the quality of life for the patients and their caregivers by reducing healthcare visits and hospital readmissions, and improving feeding habits [12].

Since publication of TOGD in 1997 [1], we have performed 66 TOGD procedures for NI patients [6], of whom 74.2% had a primary procedure and 25.8% had a rescue following a failed previous anti-reflux procedure. None of these patients have had recurrence of reflux and none have required further antireflux surgery. In view of the not uncommon difficulties surrounding redo oesophageal surgery, we have attempted to introduce a modification to the original TOGD towards a more secure oesophago-jejunal anastomosis. Of advantage during M-OGD has been the articulated 5 mm laparoscopic GIA that better fits the narrow upper quadrant of the NI patient’s abdomen and allows a safer oesophago-jejunal anastomosis. We are of the view that the retained 5 mm gastric cuff at the end of the oesophagus allows a more secure anastomosis and reduces the risk of breakdown and leakage during the immediate postoperative phase. Our three M-OGD patients have healed primarily without complications at the oesophago-jejunal anastomosis, and without internal herniae following of closure of the mesenteric spaces and the mesocolic window. This agrees with published meta-analysis where closure of both the mesenteric and Petersen defects has the lowest incidence of internal hernia following laparoscopic Roux-en-Y procedures [13].

The fragile nature of the NI patients renders time under anaesthesia of special relevance. Not all studies for laparoscopic-assisted and robotic TOGD have commented on surgical operating time [14, 15]; however, operating time for Laparoscopic TOGD has been reported up to 400 min [8]. Our three cases of M-OGD have averaged 200 min, that compares well with the 250 min for a classical TOGD [16].

We are encouraged by the success of this small experience of three cases and acknowledge that a greater number are required for strong statistical analysis. However, we believe that M-OGD has the potential to build on the reported success of TOGD, whether as a primary or a Rescue procedure, thereby improving the quality and efficiency of care of the NI patient with severe GOR.
